# Supplementary material for: On the role of parameterization in models with a misspecified nuisance component
Source: Proc Natl Acad Sci U S A. 2024 Aug 30;121(36):e2402736121. doi: 10.1073/pnas.2402736121 (PMC11388321; doi:10.1073/pnas.2402736121)
Supplement: Supplementary file 1 — Appendix 01 (PDF) [file pnas.2402736121.sapp.pdf]

PNAS

Supporting Information for

On the role of parametrization in models with a misspecified nuisance component

H. S. Battey and N. Reid

N. Reid
nancym.reid@utoronto.ca

This PDF file includes:

Supporting text
SI References

Supporting Information Text

Models and definitions. We assume that the true distribution has density function m, specified in part by the true value ψ^* of an interest parameter ψ. The model to be used for inference has likelihood function $L(\psi, \lambda)$, where ψ is the interest parameter and λ is a nuisance parameter. We assume the model is misspecified in the sense that there are no values of λ in the parameter space for the assumed model for which the true distribution is recovered. The limiting value of the maximum likelihood estimate $(\hat{\psi}, \hat{\lambda})$ obtained by maximizing the assumed likelihood function is also the value which minimizes the Kullback-Leibler divergence between the fitted model and the true distribution, and is written (ψ_m^0, λ_m^0). Our interest is in studying model structure which leads to consistency of $\hat{\psi}$, i.e. $\psi_m^0 = \psi^*$.

Definition 0.1 (symmetric parametrization)**.** Let Y_1 and Y_0 be independent random variables with probability measures in $\mathcal{P}_G$ and density functions f_1 and f_0 respectively. Their joint distribution is said to be parametrized ψ-symmetrically with respect to (ψ, γ) if $g = g_\psi \in G$ depends only on ψ and if the density functions f_1 and f_0 relate to f_U by

$$f_U(u;\gamma)du = f_1(gu; g\gamma)d(gu) = f_0(g^{-1}u; g^{-1}\gamma)d(g^{-1}u).$$

Definition 0.2 (antisymmetry)**.** The symmetric parametrization of f_1 and f_0 is said to induce antisymmetry on the associated log-likelihood derivative with respect to ψ if $\nabla_\psi \ell(\psi; \gamma, y_1, y_0)$, when expressed in terms of $u_1 = g^{-1}y_1$ and $u_0 = gy_0$, satisfies $\nabla_\psi \ell(\psi; \gamma, u_1, u_0) = -\nabla_\psi \ell(\psi; \gamma, u_0, u_1)$.

Section 1

Proposition 1.1. Let the observed log-likelihood function for the assumed model be strictly concave as a function of (ψ, λ). Then $\psi_m^0 = \psi^*$ if and only if $\mathbb{E}_m[\nabla_\psi \ell(\psi^*, \lambda_m^0)] = 0$. The latter condition is equivalent to $\mathbb{E}_m[\nabla_\psi \ell(\psi^*, \lambda)] = 0$ for all λ if and only if $\psi^* \perp_m \Lambda$.

Proof. By definition ψ_m^0 solves $\mathbb{E}_m[\nabla_{(\psi,\lambda)} \ell(\psi_m^0, \lambda_m^0)] = 0$ and therefore also $\mathbb{E}_m[\nabla_\psi \ell(\psi_m^0, \lambda_m^0)] = 0$. Taylor expansion of $\nabla_\psi \ell(\psi, \lambda_m^0)$ around ψ^* for fixed λ_m^0 followed by evaluation at ψ_m^0 gives

$$\psi_m^0 - \psi^* = -\overline{B}^{-1}\mathbb{E}_m[\nabla_\psi \ell(\psi^*, \lambda_m^0)],$$

where, by the mean value theorem for vector valued functions,

$$\overline{B} = \mathbb{E}_m\Big[\int_0^1 \nabla^2_{\psi\psi} \ell(t\psi_m^0 + (1-t)\psi^*, \lambda_m^0)dt\Big],$$

with the integral taken elementwise. Since $\overline{B}$ is positive definite, $\psi_m^0 = \psi^*$ if and only if $\mathbb{E}_m\big[\nabla_\psi \ell(\psi^*, \lambda_m^0)\big] = 0$.

A Taylor expansion of $\nabla_\psi \ell(\psi^*, \lambda)$ around λ_m^0 for fixed ψ^* shows that

$$\mathbb{E}_m[\nabla_\psi \ell(\psi^*, \lambda)] - \mathbb{E}_m[\nabla_\psi \ell(\psi^*, \lambda_m^0)] = \overline{A}(\lambda - \lambda_m^0), \qquad [1]$$

where

$$\overline{A} = \mathbb{E}_m\Big[\int_0^1 \nabla^2_{\psi\lambda} \ell(\psi^*, t\lambda + (1-t)\lambda_m^0)dt\Big] = \int_0^1 \mathbb{E}_m\big[\nabla^2_{\psi\lambda} \ell(\psi^*, t\lambda + (1-t)\lambda_m^0)\big]dt.$$

Nullity of the left hand side of Eq. (1) for general λ entails $\overline{A} = 0$, for which a sufficient condition is $\psi^* \perp_m \Lambda$. Without such orthogonality, positive and negative parts must cancel in the integral defining $\overline{A}$, which cannot be simultaneously achieved for all λ, establishing necessity of $\psi^* \perp_m \Lambda$ if $\mathbb{E}_m\big[\nabla_\psi \ell(\psi^*, \lambda)\big] = 0$ is to hold for all λ. □

Proposition 1.2. If $i^{\psi\psi}g_\psi + i^{\psi\lambda}g_\lambda = 0$ for all $\lambda \in \Lambda$, then $\psi_m^0 = \psi^*$. If ψ and λ are both scalar parameters, the condition reduces to $i_{\lambda\lambda}g_\psi = i_{\psi\lambda}g_\lambda$.

Proof. It is notationally convenient to suppose that the interest parameter ψ is scalar, although the results generalize straightforwardly to higher dimensions. We therefore write ℓ_ψ for the derivative of the log likelihood function with respect to ψ and ℓ_s for the derivative with respect to the sth component of λ, and similarly for other quantities. We use the convention that Roman letters appearing both as subscripts and superscripts in the same product are summed. Thus, for the purpose of this calculation, write $(\hat{\lambda} - \lambda)^s$ for the sth component of $\hat{\lambda} - \lambda$.

Let $\overline{\ell} = \ell/n$ and $\hat{\ell} = \overline{\ell}(\hat{\psi}, \hat{\lambda})$, etc. The rescaling by n is immaterial and is used only to aid presentation of the derivation. Taylor expansion of $\overline{\ell}_\psi$ around (ψ^*, λ) followed by evaluation at $(\hat{\psi}, \hat{\lambda})$ gives

$$\begin{aligned} 0 = \hat{\ell}_\psi \;&=\; \overline{\ell}_\psi + \overline{\ell}_{\psi\psi}(\hat{\psi} - \psi^*) + \overline{\ell}_{\psi s}(\hat{\lambda} - \lambda)^s \\ &+\; \overline{\ell}_{\psi\psi t}(\hat{\psi} - \psi^*)(\hat{\lambda} - \lambda)^t + \tfrac{1}{2}\overline{\ell}_{\psi st}(\hat{\lambda} - \lambda)^s(\hat{\lambda} - \lambda)^t + \cdots . \end{aligned}$$

Let $n\overline{j}^{rs}$ denote the components of the inverse matrix of the observed information matrix at (ψ^*, λ) whose components are $n\overline{j}_{rs} = -\ell_{rs}$. Inversion of the previous equation as in Barndorff-Nielsen and Cox (1994, p. 149) gives

$$\begin{aligned} \hat{\psi} - \psi^* \;&=\; \overline{j}^{\psi\psi}\overline{\ell}_\psi + \overline{j}^{\psi s}\overline{\ell}_s + \overline{j}^{\psi\psi}\overline{\ell}_{\psi\psi u}(\hat{\psi} - \psi^*)(\hat{\lambda} - \lambda)^u \\ &+\; \tfrac{1}{2}\overline{j}^{\psi\psi}\overline{\ell}_{\psi tu}(\hat{\lambda} - \lambda)^t(\hat{\lambda} - \lambda)^u + \tfrac{1}{2}\overline{j}^{\psi s}\overline{\ell}_{stu}(\hat{\lambda} - \lambda)^t(\hat{\lambda} - \lambda)^u + \cdots . \end{aligned} \qquad [2]$$

Write $\bar{j}^{rs} = \bar{i}^{rs} + Z_{rs}/\sqrt{n}$, where $\bar{i}^{rs}$ denotes the components of the inverse of the Fisher information matrix i_{rs} at (ψ^*, λ) rescaled by n, and Z_{rs} are random variables of zero mean and $O_p(1)$ as $n \to \infty$. Similarly write $\bar{\ell}_s = \bar{g}_s + V_s/\sqrt{n}$ where g_s is the expectation of $\bar{\ell}_s$. Expectations in $\bar{i}_{rs}$ and $\bar{g}_s$ are taken under the true model. It follows that the leading order term in the expansion is

$$\hat{\psi} - \psi^* = \bar{i}^{\psi\psi}\bar{g}_\psi + \bar{i}^{\psi s}\bar{g}_s + \bar{i}^{\psi\psi}\frac{V_\psi}{\sqrt{n}} + \bar{i}^{\psi s}\frac{V_s}{\sqrt{n}} + \frac{Z_{\psi\psi}}{\sqrt{n}}\bar{g}_\psi + \frac{Z_{\psi s}}{\sqrt{n}}\bar{g}_s + \text{Rem}.$$

If $\lambda = \lambda_m^0$, $\bar{i}^{\psi\psi}\bar{g}_\psi + \bar{i}^{\psi s}\bar{g}_s = O(n^{-1/2})$ provided that the dimension of λ is treated as fixed. This is not in general the case when $\lambda \neq \lambda_m^0$, meaning that successive replacements in equation Eq. (2) do not produce terms of decreasing orders of magnitude as in equation (5.19) of Barndorff-Nielsen and Cox (1994).

Suppose that

$$\bar{i}^{\psi\psi}\bar{g}_\psi + \bar{i}^{\psi s}\bar{g}_s = 0 \qquad [3]$$

for any λ. Again, the second and higher order terms in Eq. (2) do not converge to zero in general. The restriction to $\lambda = \lambda_m^0 + O(n^{-1/2})$ ensures that $\hat{\psi}$ converges to $\psi_m^0 = \psi^*$ at the same rate as if the model was correctly specified. But if Eq. (3) holds for all λ, it holds a fortiori for λ in a neighbourhood of λ_m^0, thereby proving the claim.

In the special case that ψ and λ are both scalar parameters, $i^{\psi\psi} = \det^{-1} i_{\lambda\lambda}$ and $i^{\psi\lambda} = -\det^{-1} i_{\psi\lambda}$, where $\det = i_{\psi\psi}i_{\lambda\lambda} - i_{\psi\lambda}^2$, so that the general condition becomes $i_{\lambda\lambda}g_\psi = i_{\psi\lambda}g_\lambda$.

□

Proposition 1.5. Suppose that the joint distribution of Y_1 and Y_0 is parametrized ψ-symmetrically with respect to (ψ, γ) in the sense of Definition 0.1. The parametrization induces antisymmetry on the log-likelihood derivative in the sense of Definition 0.2 if and only if

$$a(u_1, u_0) := \frac{\partial u_1}{\partial \psi} + \frac{\partial u_0}{\partial \psi} = -a(u_0, u_1)$$

and

$$c(u_1, u_0) := \left(\frac{\partial}{\partial \psi}\left|\frac{\partial u_1}{\partial y_1}\right|\right)\left|\frac{\partial u_0}{\partial y_0}\right| + \left(\frac{\partial}{\partial \psi}\left|\frac{\partial u_0}{\partial y_0}\right|\right)\left|\frac{\partial u_1}{\partial y_1}\right| = -c(u_0, u_1),$$

where $u_1 = g^{-1}y_1$ and $u_0 = gy_0$.

Proof. Without loss of generality, write $\nabla_u f_U(u;\gamma) = b(u;\gamma)f_U(u;\gamma)$, which may entail defining $b(u;\gamma)$ trivially as $b(u;\gamma) = \nabla_u f_U(u;\gamma)/f_U(u;\gamma)$. The form of $b(u;\gamma)$ is immaterial for the argument to be presented.

The log-likelihood derivative is antisymmetric if and only if the likelihood derivative is antisymmetric, as $\nabla_\psi \ell = \nabla_\psi L/L$ and L is symmetric in u_1 and u_0 by Definition 0.1. Hence consider

$$\nabla_\psi L(\psi;\gamma, y_1, y_0) = f_0(y_0; g^{-1}\gamma)\nabla_\psi f_1(y_1; g\gamma) + f_1(y_1; g\gamma)\nabla_\psi f_0(y_0; g^{-1}\gamma) \qquad [4]$$

where, on writing $u_1 = g^{-1}y_1$ and $u_0 = gy_0$,

$$\nabla_\psi f_1(y_1; g\gamma) = \left|\frac{\partial u_1}{\partial y_1}\right|\frac{\partial}{\partial \psi}f_U(g^{-1}y_1;\gamma) + f_U(g^{-1}y_1;\gamma)\frac{\partial}{\partial \psi}\left|\frac{\partial u_1}{\partial y_1}\right|$$

and

$$\frac{\partial}{\partial \psi}f_U(g^{-1}y_1;\gamma) = \frac{\partial}{\partial u_1}f_U(u_1;\gamma)\frac{\partial u_1}{\partial \psi} = b(u_1;\gamma)f_U(u_1;\gamma)\frac{\partial u_1}{\partial \psi}.$$

Analogous expressions hold for $\nabla_\psi f_0(y_0; g^{-1}\gamma)$, giving

$$\nabla_\psi f_1(y_1; g\gamma) = f_U(u_1;\gamma)\left(b(u_1;\gamma)\frac{\partial u_1}{\partial \psi}\left|\frac{\partial u_1}{\partial y_1}\right| + \frac{\partial}{\partial \psi}\left|\frac{\partial u_1}{\partial y_1}\right|\right)$$

and

$$\nabla_\psi f_0(y_0; g^{-1}\gamma) = f_U(u_0;\gamma)\left(b(u_0;\gamma)\frac{\partial u_0}{\partial \psi}\left|\frac{\partial u_0}{\partial y_0}\right| + \frac{\partial}{\partial \psi}\left|\frac{\partial u_0}{\partial y_0}\right|\right)$$

respectively. On substituting in Eq. (4) and expressing the resulting quantities in terms of u_1 and u_0 we obtain

$$\nabla_\psi L(\psi;\gamma, u_1, u_0) = f_U(u_1;\gamma)f_U(u_0;\gamma)B(u_1, u_0)$$

with

$$B(u_1, u_0) = \left(b(u_1;\gamma)\frac{\partial u_1}{\partial \psi}\left|\frac{\partial u_1}{\partial y_1}\right| + \frac{\partial}{\partial \psi}\left|\frac{\partial u_1}{\partial y_1}\right|\right)\left|\frac{\partial u_0}{\partial y_0}\right| + \left(b(u_0;\gamma)\frac{\partial u_0}{\partial \psi}\left|\frac{\partial u_0}{\partial y_0}\right| + \frac{\partial}{\partial \psi}\left|\frac{\partial u_0}{\partial y_0}\right|\right)\left|\frac{\partial u_1}{\partial y_1}\right|.$$

so that the parametrization induces antisymmetry on the likelihood derivative in the sense of Definition 0.2 if and only if $B(u_1, u_0) = -B(u_0, u_1)$, which is seen to be equivalent to the conditions above on noting that $J_1^+ J_0^+ = 1$ by the symmetry of the parametrization.

□

Section 2

Proposition 2.1. Suppose that the distribution over γ is parametrized by λ, producing a log-likelihood contribution $\ell(\psi, \lambda; y_1, y_0)$, assumed strictly concave. Suppose further that, conditionally on γ, Y_1 and Y_0 have a joint distribution that is parametrized ψ-symmetrically (Definition 0.1). Then provided that the group induces antisymmetry on the log-likelihood derivative (Definition 0.2), it follows that $\psi^* \perp_m \Lambda$ and

$$0 = \mathbb{E}_m[\nabla_\psi \ell(\psi^*, \lambda)] = \int_{\mathscr{Y}} \int_{\mathscr{Y}} \big(\nabla_\psi \ell(\psi^*, \lambda; y_1, y_0)\big) m(y_1, y_0) dy_1 dy_0 \qquad [5]$$

for all $\lambda \in \Lambda$. Thus, $\psi_m^0 = \psi^*$ by Proposition 1.1.

Proof. In Eq. (5),

$$m(y_1, y_0) = \int f_1(y_1; \psi^*, \gamma) f_0(y_0; \psi^*, \gamma) f(\gamma) d\gamma \qquad [6]$$

with $f(\gamma)$ an unknown density function for the nuisance parameter. All quantities are, for present purposes, evaluated at the same value of ψ and the superscript is henceforth suppressed.

Equation 5 is established first. Since $\nabla_\psi \ell(\psi, \lambda)$ does not depend on γ the order of integration in Eq. (5) can be interchanged, giving the sufficient condition

$$\int_{\mathscr{Y}} \int_{\mathscr{Y}} \big(\nabla_\psi \ell(\psi, \lambda; y_1, y_0)\big) f_1(y_1; \psi, \gamma) f_0(y_0; \psi, \gamma) dy_1 dy_0 = 0 \qquad [7]$$

identically in γ. Suppose that, conditionally on γ, Y_1 and Y_0 have a joint distribution that is parametrized symmetrically in the sense of Definition 0.1. On the left hand side of Eq. (7), change variables to $u_1 = g^{-1} y_1$ and $u_0 = g y_0$. The symmetry of the parametrization gives $J_1^+ J_0^+ = 1$ in Definition 0.1 and similarly $(\partial u_1/\partial y_1)^{-1}(\partial u_0/\partial y_0)^{-1} = 1$. Therefore the volume element satisfies $dy_1 dy_0 = du_1 du_0$ and Eq. (7) is

$$\int_{\mathscr{Y}} \int_{\mathscr{Y}} \nabla_\psi \ell(\psi, \lambda; u_1, u_0) f_U(u_1; \gamma) f_U(u_0; \gamma) du_1 du_0 = 0, \qquad [8]$$

where the limits of integration are unaltered as a result of the assumed group structure. Since $f_U(u_1;\gamma) f_U(u_0;\gamma) du_1 du_0$ is symmetric in u_1 and u_0 and since f_U is non-negative, a necessary and sufficient condition for Eq. (8) is antisymmetry of $\nabla_\psi \ell(\psi, \lambda; u_1, u_0)$, in the sense

$$\nabla_\psi \ell(\psi, \lambda; u_1, u_0) = -\nabla_\psi \ell(\psi, \lambda; u_0, u_1). \qquad [9]$$

Here the derivative with respect to ψ is taken first, followed by evaluation at $u_1 = g^{-1} y_1$ and $u_0 = g y_0$.

Next consider the orthogonality condition of Proposition 1.1. The global orthogonality equation in the matched comparison problem is

$$\int_{\mathscr{Y}} \int_{\mathscr{Y}} \nabla^2_{\psi\lambda} \ell(\psi, \lambda; y_1, y_0) m(y_1, y_0) dy_1 dy_0 = 0,$$

where $m(y_1, y_0) = m(y_1, y_0; \psi^*)$ is as defined in Eq. (6) and ψ^* has been reintroduced for clarity. Since m does not depend on λ,

$$\int_{\mathscr{Y}} \int_{\mathscr{Y}} \nabla^2_{\psi\lambda} \ell(\psi, \lambda; y_1, y_0) m(y_1, y_0) dy_1 dy_0 = \nabla_\lambda \int_{\mathscr{Y}} \int_{\mathscr{Y}} \nabla_\psi \ell(\psi, \lambda; y_1, y_0) m(y_1, y_0; \psi^*) dy_1 dy_0.$$

The double integral on the right hand side is, apart from the evaluation at ψ^*, that of Eq. (5). Orthgonality at ψ^* thus follows by an identical argument to that establishing Eq. (5). The orthogonality is therefore local at ψ^* and global in λ, as required in Proposition 1.1.

The log-likelihood contribution from a single pair, treated as a function of both (ψ, λ) and (y_1, y_0) is

$$\ell(\psi, \lambda; y_1, y_0) = \log \int f_1(y_1; \psi, \gamma) f_0(y_0; \psi, \gamma) h(\gamma; \lambda) d\gamma = \log L(\psi, \lambda; y_1, y_0),$$

say. The same argument as above shows that the likelihood function $L(\psi, \lambda; y_1, y_0)$ is symmetric when expressed in terms of $u_1 = g^{-1} y_1$ and $u_0 = g y_0$. Since $\nabla_\psi \ell = \nabla_\psi L / L$, the antisymmetry condition Eq. (9) is satisfied if $\nabla_\psi L$ is antisymmetric. The latter is implied by the clause of Proposition 2.1 as

$$\nabla_\psi L(\psi, \lambda; y_1, y_0) = \int \nabla_\psi L(\psi; \gamma, y_1, y_0) h(\gamma; \lambda) d\gamma.$$

□

Proposition 2.2. Suppose that, conditionally on γ, S_1 and S_0 are independent random variables with probability measures in $\mathcal{P}_G$. Let $h_1 \in G$ and $h_0 \in G$, not depending on ψ or γ be such that $T_1 = h_1 S_1$ and $T_0 = h_0 S_0$, with density functions f_1 and f_0 with respect to Lebesgue measure, have a joint distribution that is parametrized ψ-symmetrically in the sense of Definition 0.1 and such that the log-likelihood derivative, when expressed in terms of $u_1 = g^{-1}t_1$ and $u_0 = gt_0$, is antisymmetric in the sense of Definition 0.2. Then, provided that $\ell(\psi, \lambda)$ is strictly concave, $\psi^* \perp \Lambda$ and

$$0 = \mathbb{E}_m[\nabla_\psi \ell(\psi^*, \lambda)] = \int\int \big(\nabla_\psi \ell(\psi^*, \lambda; s_1, s_0)\big) m(s_1, s_0) ds_1 ds_0 \qquad [10]$$

for all $\lambda \in \Lambda$. Thus, $\psi_m^0 = \psi^*$ by Proposition 1.1.

Proof. The analogue of equation Eq. (7) in the proof of Proposition 2.1 is

$$\int_{\mathscr{Y}}\int_{\mathscr{Y}} \nabla_\psi \ell(\psi, \lambda; s_1, s_0) f_1(s_1; \psi, \gamma) f_0(s_0; \psi, \gamma) ds_1 ds_0 = 0 \qquad [11]$$

identically in γ. In the likelihood derivative Eq. (4), the relevant terms are

$$\nabla_\psi f_1(s_1; h_1 g\gamma) = \left|\frac{\partial t_1}{\partial s_1}\right| \nabla_\psi f_{T_1}(t_1; g\gamma), \qquad \nabla_\psi f_0(s_0; h_0 g^{-1}\gamma) = \left|\frac{\partial t_0}{\partial s_0}\right| \nabla_\psi f_{T_0}(t_0; g^{-1}\gamma),$$

and similarly for $f_1(s_1; h_1 g\gamma)$ and $f_0(s_0; h_0 g^{-1}\gamma)$, where $t_1 = h_1 s_1$, $t_0 = h_0 s_0$ and the maps $h_1, h_0 \in G$ do not depend on ψ or γ. Thus the relevant analogue of Eq. (4) is

$$\left|\frac{\partial t_1}{\partial s_1}\right| \left|\frac{\partial t_0}{\partial s_0}\right| \nabla_\psi L(\psi; \gamma, t_1, t_0)$$

and the sufficient condition Eq. (11) reduces to

$$\int_{\mathscr{Y}}\int_{\mathscr{Y}} \nabla_\psi \ell(\psi, \lambda; t_1, t_0) f_{T_1}(t_1; \psi, \gamma) f_{T_0}(t_0; \psi, \gamma) dt_1 dt_0 = 0.$$

Since T_1 and T_0 have a joint distribution that is parametrized ψ-symmetrically with respect to (ψ, γ), and such that the log-likelihood derivative, when expressed in terms of $u_1 = g^{-1}t_1$ and $u_0 = gt_0$, is antisymmetric, both conditions of Proposition 1.1 hold by the proofs of Propositions 1.4 and 2.1.

□

Proposition 2.3. [Cox and Wong, (1)] Suppose that in Example 2.3, the nuisance parameters $(\gamma_j)_{j=1}^n$ are modelled as independent and identically distributed random variables with gamma density $\xi(\xi\gamma)^{\omega-1}\exp(-\xi\gamma)/\Gamma(\omega)$, parametrized in terms of the notionally orthogonal parameters $\nu = \xi/\omega$ and $\omega > 0$. Provided that $(\gamma_j)_{j=1}^n$ have the same expectation $1/\nu$ under the true and assumed random effects distribution, $\hat{\theta}$ is consistent for θ^*.

Proof. Let $S_j = Y_{j0} + Y_{j1}$ and $D_j = Y_{j1} - Y_{j0}$ with realizations s_j, d_j. Using the notation of Cox and Wong, further define $r_j(\theta) = r_{j0}e^{-\theta} + r_{j1}e^{\theta}$, $r_j(\theta, \nu\omega) = r_j(\theta) + \nu\omega$. The contribution of the jth stratum to the log-likelihood function is

$$\log\{\Gamma(s_j + \omega)/\Gamma(\omega)\} + \theta d_j + \omega\log\omega + \omega\log\nu - (s_j + \omega)\log r_j(\theta, \nu\omega)$$

and the log-likelihood function ℓ is a sum of m such contributions. On noting that $\mathbb{E}_{\theta,\nu,\omega}(S_j) = r_j(\theta)\mathbb{E}_{\nu,\omega}(\gamma_j) = r_j(\theta)/\nu$ under the assumed model, direct calculation shows that the Fisher information under the gamma random effects assumption has components $\check{\imath}_{\theta\omega} = 0 = \check{\imath}_{\nu\omega}$ identically in (θ, ν, ω), implying by the clause of Proposition 2.3 that $\Theta \perp_m \Omega$, $\mathcal{N} \perp_m \Omega$, where Θ, Ω and $\mathcal{N}$ are the parameter spaces for θ, ω and ν. The parameter ω does not therefore play a role, and the problem can be treated as though ω were known.

On noting that $\mathbb{E}(D_j) = \nabla_\theta r_j(\theta)/\nu$

$$\begin{aligned}
\mathbb{E}_m(\nabla_\theta \ell) = \mathbb{E}_{\theta,\nu,\omega}(\nabla_\theta \ell) &= \sum_j \left(\frac{\nabla_\theta r_j(\theta)}{\nu} - \frac{\{r_j(\theta)/\nu + \omega\}\nabla_\theta r_j(\theta)}{r_j(\theta, \nu\omega)}\right) = 0, \\
\mathbb{E}_m(\nabla_\nu \ell) = \mathbb{E}_{\theta,\nu,\omega}(\nabla_\nu \ell) &= \sum_j \left(\frac{\omega}{\nu} - \frac{\{r_j(\theta)/\nu + \omega\}\omega}{r_j(\theta, \nu\omega)}\right) = 0,
\end{aligned}$$

verifying the weaker condition of Proposition 1.2.

□

Section 3

Proposition 3.1. Suppose that, conditional on observed explanatory features, the probability density or mass function of $Y=(Y_1,\ldots,Y_n)$ at $y=(y_1,\ldots,y_n)$ is of canonical exponential family regression form, that is,

$$m(y;x_1^{\mathrm{T}}\psi^*,\ldots,x_n^{\mathrm{T}}\psi^*)=\exp\Big\{\sum_{i=1}^n y_i x_i^{\mathrm{T}}\psi^* - \sum_{i=1}^n K(x_i^T\psi^*)\Big\}\prod_{i=1}^n h(y_i).$$

Suppose further that the analysis is overstratified so that under the assumed model the log-likelihood function is $\ell(\psi,\lambda)=s^{\mathrm{T}}\psi+t^{\mathrm{T}}\lambda-K(\psi,\lambda)$, where $s=\sum_{i=1}^n x_i y_i$, $t=\sum_{i=1}^n w_i y_i$ and $K(\psi,\lambda)=\sum_{i=1}^n K(x_i^{\mathrm{T}}\psi+w_i^{\mathrm{T}}\lambda)$. The conditions of Propositions 1.1 and 1.2 are in general violated, yet the maximum likelihood estimator $\hat{\psi}$ is consistent for ψ^*.

Proof. Index the expectation under the overstratified model by $\xi=(\xi_1,\ldots,\xi_n)=(x_1^{\mathrm{T}}\psi+w_1^{\mathrm{T}}\lambda,\ldots,x_n^{\mathrm{T}}\psi+w_n^{\mathrm{T}}\lambda)$ with $\xi^*=(x_1^{\mathrm{T}}\psi^*+w_1^{\mathrm{T}}\lambda,\ldots,x_n^{\mathrm{T}}\psi^*+w_n^{\mathrm{T}}\lambda)$ and true value $\eta=(\eta_1,\ldots,\eta_n)=(x_1^{\mathrm{T}}\psi^*,\ldots,x_n^{\mathrm{T}}\psi^*)$. Then, m is synonymous with η and we write $\mathbb{E}_m=\mathbb{E}_\eta$. With $g_\psi=\mathbb{E}_\eta[\nabla_\psi\ell(\psi^*,\lambda)]$ and $g_\lambda=\mathbb{E}_\eta[\nabla_\lambda\ell(\psi^*,\lambda)]$,

$$\begin{aligned} g_\psi &= \mathbb{E}_\eta(S)-\sum_{i=1}^n\nabla_\psi K(x_i^{\mathrm{T}}\psi+w_i^{\mathrm{T}}\lambda)=\sum_{i=1}^n x_i d_i \\ g_\lambda &= \mathbb{E}_\eta(T)-\sum_{i=1}^n\nabla_\lambda K(x_i^{\mathrm{T}}\psi+w_i^{\mathrm{T}}\lambda)=\sum_{i=1}^n w_i d_i, \end{aligned}$$

where $d_i=K'(\eta_i)-K'(\xi_i^*)$. On letting $D^*=\mathrm{diag}(K''(\xi_1^*),\ldots,K''(\xi_n^*))$, the components of the information matrix i at (ψ^*,λ) are $i_{\psi\psi}=\sum_{i=1}^n K''(\xi_i^*)x_i x_i^{\mathrm{T}}=X^{\mathrm{T}}D^*X$, $i_{\lambda\lambda}=\sum_{i=1}^n K''(\xi_i^*)w_i w_i^{\mathrm{T}}=W^{\mathrm{T}}D^*W$, $i_{\psi\lambda}=\sum_{i=1}^n K''(\xi_i^*)x_i w_i^{\mathrm{T}}=X^{\mathrm{T}}D^*W$ and $i_{\lambda\psi}=\sum_{i=1}^n K''(\xi_i^*)w_i x_i^{\mathrm{T}}=W^{\mathrm{T}}D^*X$.

The relevant components of i^{-1} from Proposition 1.2 are

$$\begin{aligned} i^{\psi\psi} &= (i_{\psi\psi}-i_{\psi\lambda}i_{\lambda\lambda}^{-1}i_{\lambda\psi})^{-1} \\ i^{\psi\lambda} &= -(i_{\psi\psi}-i_{\psi\lambda}i_{\lambda\lambda}^{-1}i_{\lambda\psi})^{-1}i_{\psi\lambda}i_{\lambda\lambda}^{-1}=i^{\psi\psi}i_{\psi\lambda}i_{\lambda\lambda}^{-1} \end{aligned}$$

If derivatives of K higher than 2 are null, as for linear regression, $d=D^*W\lambda$ by a Taylor expansion of $K'(\eta)$ so that $i^{\psi\psi}g_\psi=i^{\psi\psi}X^{\mathrm{T}}D^*W\lambda$ and $i^{\psi\lambda}g_\lambda=-i^{\psi\psi}X^{\mathrm{T}}D^*W\lambda$, verifying Proposition 1.2. More generally however, $d\neq D^*W\lambda$ and the consistency arises instead because $\lambda_m^0=\lambda^*=0$, at which point the condition is trivially satisfied.

□

References

1. DR Cox, MY Wong, A note on the sensitivity of assumptions of a generalized linear mixed model. *Biometrika* **97**, 209–214 (2010).
